# Mitochondrial genomes of the freshwater sponges *Spongilla lacustris* and *Ephydatia cf. muelleri*

**DOI:** 10.1080/23802359.2016.1157771

**Published:** 2016-03-29

**Authors:** Warren Russell Francis, Michael Eitel, Sergio Vargas, Stefan Krebs, Helmut Blum, Gert Wörheide

**Affiliations:** a Department of Earth and Environmental Sciences, Paleontology & Geobiology, Ludwig-Maximilians-Universität München, Munich, Germany;; b Laboratory for Functional Genome Analysis (LAFUGA), Gene Center, Ludwig-Maximilians-Universität München, Munich, Germany;; c GeoBio-Center, Ludwig-Maximilians-Universität München, Munich, Germany;; d Bavarian State Collections for Paleontology and Geology, Munich, Germany

**Keywords:** Freshwater, porifera, sponge, sympatric

## Abstract

We report the mitochondrial genomes of two freshwater sponges, *Spongilla lacustris* and *Ephydatia cf. muelleri*. The genomes contain 14 protein-coding genes and are similar in structure to other published mitochondrial genomes from freshwater sponges. The *E. cf. muelleri* described here is remarkably similar in coding regions to the published genome, but differs in number and length of hairpin-forming repeats between genes.

Sponges are a diverse animal phylum containing an estimated 15,000 species that perform important physiological and ecological roles in aquatic ecosystems. While the majority of species inhabit marine environments, members of the order Spongillida are found in freshwater habitats around the world. Previous analysis of the mitochondrial protein CO1 suggested that freshwater sponges originated from a single, relatively recent radiation (Itskovich et al. 2006). Here we report the complete mitochondrial (mt) genomes of two freshwater demosponges from Lake Constance in Germany, *Spongilla lacustris* – the type genus and species of the largest family of freshwater sponges, and the co-sequenced *Ephydatia cf. muelleri var. bodenseensis*.

We sequenced the genome of *Spongilla lacustris* gemmules using the Illumina Moleculo (TruSeq) artificial long-read technology. The long reads were mapped using Kearse et al. (2012) to the published mt genome of *Ephydatia muelleri* (Wang & Lavrov 2008) allowing for indels and up to 20% mismatch. Mapped reads were then assembled using the built-in assembler in Geneious R8, producing a single contig of 28,044 bases with a coverage of 317×. The published *cox1* gene from *S. lacustris* (EU000572.1) matched to the assembled version with 99% identity (two substitutions), confirming the species. From the mapping of the long reads, we found reads with unusually high identity (99%) to the published mt sequence of *E. muelleri*. These reads were separated and assembled into a distinct mt genome of 23,946 bp at 41× coverage with 99% identity to the published *E. muelleri*. This finding suggests that *S. lacustris* and *E. muelleri* occur sympatrically and are almost indistinct to divers.

Gene annotation was done using the MFannot web server (Valach et al. 2014). Both genomes contain the same 14 protein-coding genes, 25 tRNAs and two ribosomal RNAs, all in the same order as all other published freshwater sponges: *Lubomirskia baicalensis* (Lavrov 2010), *Rezinkovia echinata*, *Baikalospongia intermedia*, *Swartschewskia papyracea*, *Corvomeyenia sp*. (Lavrov et al. 2012), *Eunapius subterraneus* (Plese et al. 2012) and *Ephydatia fluviatilis* (Imesek et al. 2013).

Among the species compared, the lowest sequence identities were 68.2% between *S. lacustris* and both *L. baicalensis* and *B. intermedia*. When comparing only the coding sequences, the minimum sequence identity was 97.7% between *S. lacustris* and *Corvomeyenia sp*. Within the entire clade, the lowest coding sequence identity was 96.4% between *Corvomeyenia sp*. and *E. fluviatilis*, showing that sequence divergence is low in this order (Figure 1). The *E. cf. muelleri var bodenseensis* differs from the published *E. muelleri* by only three nucleotides substitutions within the coding regions and one nucleotide substitution in trnT, while the overall identity is 99.4% between the two variants. The intergenic regions vary considerably with most of the differences occurring as insertions of repetitive hairpin-forming elements (RHEs), as seen in other sponges (Erpenbeck et al. 2009; Lavrov et al. 2012).

**Figure 1.**
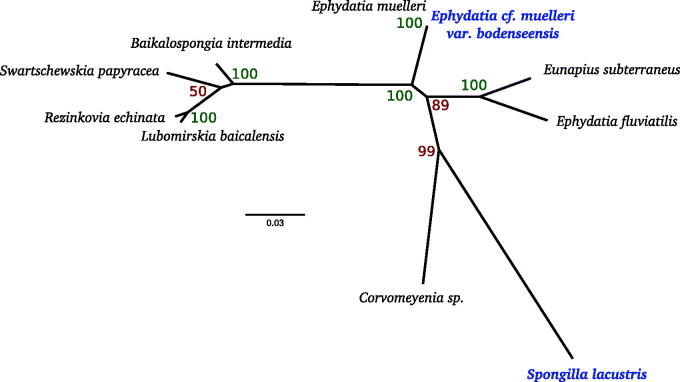
Phylogenetic tree of mitochondrial sequences from freshwater sponges. Whole mitochondrial genomic nucleotide sequences were used for all species. Alignment was made with MAFFT using LINSI parameters, and tree was generated using RAXML 8.2 (Exelixis Lab, Tucson, AZ) using the GTRGAMMA model with 100 bootstrap replicates. Species in bold were sequenced in this study. Species accessions are as follows: *L. baicalensis*, NC_013760.1; *R. echinata*, NC_018360.1; *B. intermedia*, NC_018343.1; *S. papyracea*, JQ302308; *Corvomeyenia sp*., JQ302311; *E. subterraneus*, NC_016431.1; *E. muelleri*, NC_010202.1; *E. fluviatilis*, JN209966.

This Whole Genome Shotgun project has been deposited in ENA under the accession numbers LT158503 and LT158504. Specimens and live cultures are kept at the Department of Earth and Environmental Sciences, Paleontology & Geobiology, Ludwig-Maximilians-Universität München.
